# Fried Apples

**Published:** 1854-12

**Authors:** 


FRIED APPLES.—Wash them, cut in two, remove the stem and core, and unpealed put them into a pan with butter and some water, put on the lid, place them on a stove, stir them now and then until they are soft, but do not burn them. Sour apples do not fry well.

